# Supplementary material for: Hotspots of human mutation point to clonal expansions in spermatogonia
Source: Nature. 2025 Oct 8;647(8089):429–35. doi: 10.1038/s41586-025-09579-7 (PMC12714578; doi:10.1038/s41586-025-09579-7)
Supplement: Supplementary file 2 — Reporting Summary [file 41586_2025_9579_MOESM2_ESM.pdf]

nature portfolio

Corresponding author(s): Shamil Sunyaev, Mikhail A. Moldovan, Vladimir Seplyarskiy

Last updated by author(s): Aug 19, 2025

Reporting Summary

Nature Portfolio wishes to improve the reproducibility of the work that we publish. This form provides structure for consistency and transparency in reporting. For further information on Nature Portfolio policies, see our Editorial Policies and the Editorial Policy Checklist.

Statistics

For all statistical analyses, confirm that the following items are present in the figure legend, table legend, main text, or Methods section.

n/a	Confirmed	
☐	☒	The exact sample size (*n*) for each experimental group/condition, given as a discrete number and unit of measurement
☒	☐	A statement on whether measurements were taken from distinct samples or whether the same sample was measured repeatedly
☐	☒	The statistical test(s) used AND whether they are one- or two-sided<br>*Only common tests should be described solely by name; describe more complex techniques in the Methods section.*
☒	☐	A description of all covariates tested
☐	☒	A description of any assumptions or corrections, such as tests of normality and adjustment for multiple comparisons
☐	☒	A full description of the statistical parameters including central tendency (e.g. means) or other basic estimates (e.g. regression coefficient) AND variation (e.g. standard deviation) or associated estimates of uncertainty (e.g. confidence intervals)
☐	☒	For null hypothesis testing, the test statistic (e.g. *F*, *t*, *r*) with confidence intervals, effect sizes, degrees of freedom and *P* value noted<br>*Give P values as exact values whenever suitable.*
☒	☐	For Bayesian analysis, information on the choice of priors and Markov chain Monte Carlo settings
☒	☐	For hierarchical and complex designs, identification of the appropriate level for tests and full reporting of outcomes
☐	☒	Estimates of effect sizes (e.g. Cohen's *d*, Pearson's *r*), indicating how they were calculated

Our web collection on statistics for biologists contains articles on many of the points above.

Software and code

Policy information about availability of computer code

Data collection	No software for data collection has been used
Data analysis	For data analysis, we used custom R and Python code that is deposited online under the MIT license at: https://github.com/mikemoldovan/CES_Discovery

For manuscripts utilizing custom algorithms or software that are central to the research but not yet described in published literature, software must be made available to editors and reviewers. We strongly encourage code deposition in a community repository (e.g. GitHub). See the Nature Portfolio guidelines for submitting code & software for further information.

Data

Policy information about availability of data

All manuscripts must include a data availability statement. This statement should provide the following information, where applicable:

- Accession codes, unique identifiers, or web links for publicly available datasets
- A description of any restrictions on data availability
- For clinical datasets or third party data, please ensure that the statement adheres to our policy

We used the following publicly available datasets:
Genetic variation in the human population reported in gnomAD-v4: https://gnomad.broadinstitute.org/news/2023-11-gnomad-v4-0/

Datasets of de novo variants in cohorts of patients affected with a variety conditions published as supplementary information in Refs 9,12,13,15,19,50-54
Sperm sequencing dataset published in the preprint by Neville et al., 2024 (DOI: 10.1101/2024.10.30.24316414v1)

Research involving human participants, their data, or biological material

Policy information about studies with human participants or human data. See also policy information about sex, gender (identity/presentation), and sexual orientation and race, ethnicity and racism.

Reporting on sex and gender	N/A
Reporting on race, ethnicity, or other socially relevant groupings	N/A
Population characteristics	N/A
Recruitment	N/A
Ethics oversight	N/A

Note that full information on the approval of the study protocol must also be provided in the manuscript.

Field-specific reporting

Please select the one below that is the best fit for your research. If you are not sure, read the appropriate sections before making your selection.

☒ Life sciences ☐ Behavioural & social sciences ☐ Ecological, evolutionary & environmental sciences

For a reference copy of the document with all sections, see nature.com/documents/nr-reporting-summary-flat.pdf

Life sciences study design

All studies must disclose on these points even when the disclosure is negative.

Sample size	Numbers of samples in the used datasets are as reported in the original studies
Data exclusions	We applied Roulette variant-quality filters (described in Ref 24) to censor positions with low quality of mutation rate predictions. Otherwise, no data were excluded.
Replication	As our study is theoretical in nature, experimental replication is not applicable here. To ensure computational reproducibility, we have created a GitHub repository containing all the relevant code (as stated in the "data availability statement" and "code availability statement")
Randomization	As no experiments were designed, no randomization procedures were implemented. Nor was there any randomized subsampling of the repurposed data and no random bootstrap- permutation-based statistical tests which could be considered a form of randomization.
Blinding	As no human decision-based procedures were employed, blinding is not applicable in this case.

Reporting for specific materials, systems and methods

We require information from authors about some types of materials, experimental systems and methods used in many studies. Here, indicate whether each material, system or method listed is relevant to your study. If you are not sure if a list item applies to your research, read the appropriate section before selecting a response.

Materials & experimental systems

n/a	Involved in the study	
☒	☐	Antibodies
☒	☐	Eukaryotic cell lines
☒	☐	Palaeontology and archaeology
☒	☐	Animals and other organisms
☒	☐	Clinical data
☒	☐	Dual use research of concern
☒	☐	Plants

Methods

n/a	Involved in the study	
☒	☐	ChIP-seq
☒	☐	Flow cytometry
☒	☐	MRI-based neuroimaging

Plants

Seed stocks	N/A
Novel plant genotypes	N/A
Authentication	N/A
