# Supplementary figures and images for: Hotspots of human mutation point to clonal expansions in spermatogonia
Source: Nature. 2025 Oct 8;647(8089):429–35. doi: 10.1038/s41586-025-09579-7 (PMC12714578; doi:10.1038/s41586-025-09579-7)

% cells expressing a gene vs. geneID

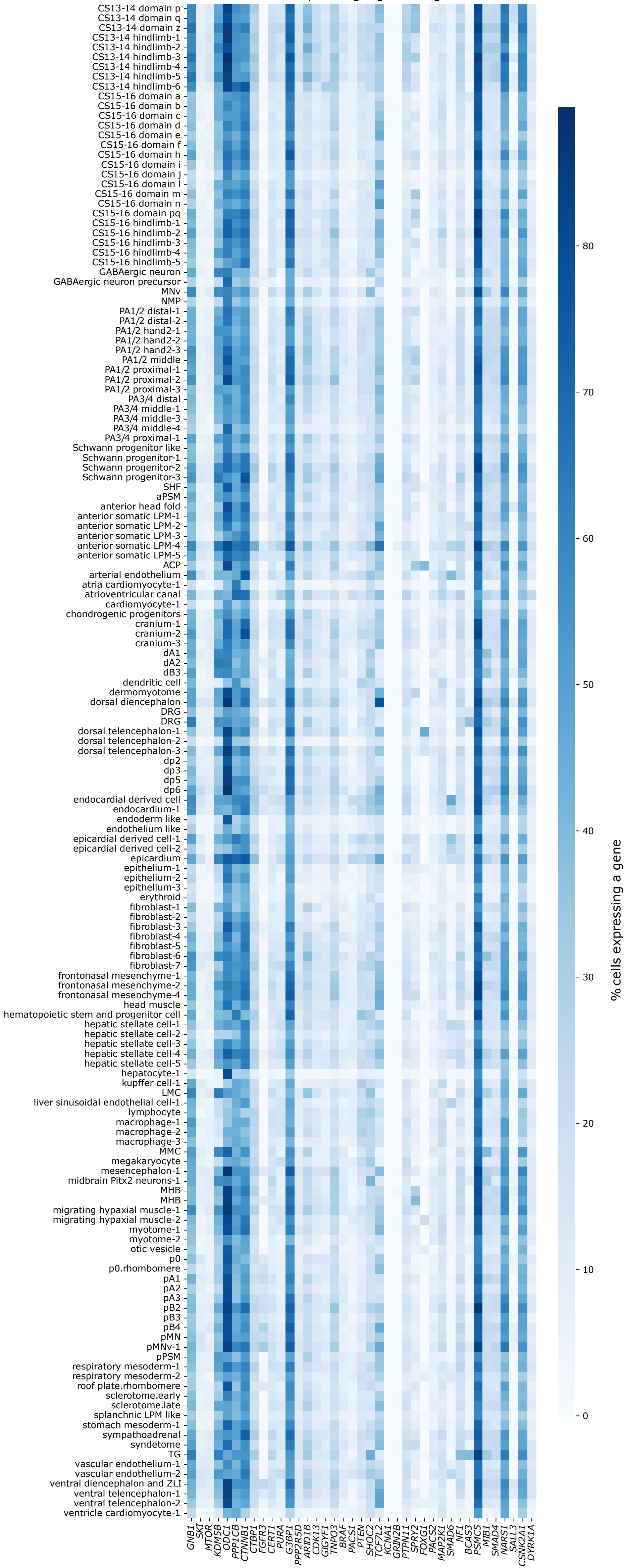

Supplement: Supplementary file 3 — Heatmap of expression of identified CES drivers in fetal single-cell expression clusters identified by Xu et al. (ref. 57). [file 41586_2025_9579_MOESM3_ESM.pdf]
